# Cyanamide Potentiates the Ethanol-Induced Impairment of Receptor-Mediated Endocytosis in a Recombinant Hepatic Cell Line Expressing Alcohol Dehydrogenase Activity

**DOI:** 10.1155/2012/954157

**Published:** 2012-02-14

**Authors:** Dahn L. Clemens, Dean J. Tuma, Carol A. Casey

**Affiliations:** ^1^Liver Study Unit, Department of Veterans' Affairs, Omaha, NE 68105, USA; ^2^Department of Internal Medicine, University of Nebraska Medical Center, Omaha, NE 68198, USA

## Abstract

Ethanol administration has been shown to alter receptor-mediated endocytosis in the liver. We have developed a recombinant hepatic cell line stably transfected with murine alcohol dehydrogenase cDNA to serve as an *in vitro* model to investigate these ethanol-induced impairments. In the present study, transfected cells were maintained in the absence or presence of 25 mM ethanol for 7 days, and alterations in endocytosis by the asialoglycoprotein receptor were determined. The role of acetaldehyde in this dysfunction was also examined by inclusion of the aldehyde dehydrogenase inhibitor, cyanamide. Our results showed that ethanol metabolism impaired internalization of asialoorosomucoid, a ligand for the asialoglycoprotein receptor. The addition of cyanamide potentiated the ethanol-induced defect in internalization and also impaired degradation of the ligand in the presence of ethanol. These results indicate that the ethanol-induced impairment in endocytosis is exacerbated by the inhibition of aldehyde dehydrogenase, suggesting the involvement of acetaldehyde in this dysfunction.

## 1. Introduction

Previous work from our laboratories has shown that ethanol administration impairs multiple aspects of the process of receptor-mediated endocytosis (RME) in isolated hepatocytes [1–8]. Decreased binding, internalization, and degradation of two ligands known to be internalized by RME, asialoorosomucoid (ASOR) and epidermal growth factor (EGF), have been described. These impairments appear to be due to the metabolism, and not to the acute presence, of ethanol. However, the direct involvement of ethanol metabolism in these impairments has yet to be demonstrated. In order to study these impairments in more detail, we have developed a cell culture system. Using the differentiated hepatoblastoma cell line Hep G2, which does not efficiently metabolize ethanol, we have established a stably transfected cell line that expresses alcohol dehydrogenase activity [9]. Hep G2 cells were chosen since previous work has shown that the cells actively bind, internalize, and degrade ASOR by RME in a similar fashion to that which is seen with rat hepatocytes [10]. These cells, designated HAD (for having alcohol dehydrogenase), have previously been shown to metabolize ethanol efficiently and produce physiological amounts of acetaldehyde. In addition, impaired binding of the ligand, ASOR, to the asialoglycoprotein receptor has been demonstrated in these cells after chronic incubation with ethanol [11]. The impaired ligand binding was alleviated by the addition of pyrazole (an alcohol dehydrogenase inhibitor). These results indicated that the impairment in binding of ASOR was dependent on the oxidation of ethanol, providing very strong evidence for a requirement of ethanol metabolism in this dysfunction. In the current study, we have further characterized the impaired RME by investigating the effects of 7 days of ethanol administration on internalization, degradation, and binding of ASOR in HAD cells. We also incubated cells in the presence of ethanol and cyanamide (an aldehyde dehydrogenase inhibitor), to increase steady-state levels of acetaldehyde in the cultures in order to examine the involvement of acetaldehyde in the impairments.

## 2. Materials and Methods

### 2.1. Establishment of Recombinant HAD Cells

HepG2 cells were stably transfected with pIC-14, an eukaryotic expression plasmid containing a cDNA copy of the murine alcohol dehydrogenase gene *Adh-1* as described in a previous paper [9].

### 2.2. Cells and Culture Conditions

Hep G2 cells [12] were cultured in Dulbecco's modified Eagle's medium (DMEM) containing high glucose supplemented with 2 mM L-glutamine, 10% fetal bovine serum, and 50 ug/mL gentamicin. Recombinant HAD cells were cultured in the same medium containing 200 ug/mL hygromycin B. All cells were cultured at 37°C for 7 days in a humidified environment containing 5% CO_2_. During ethanol and acetaldehyde metabolism studies, the growth media were supplemented with 25 mM ethanol or 0.1 mM cyanamide (or both) and were culture in sealed 25 cm^2^ flasks to minimize evaporation of ethanol and acetaldehyde.

### 2.3. Binding of ASOR and Antiasialoglycoprotein Receptor (ASGP-R) Antibody to Intact Cells

Cells were removed from the plates with Eagle's medium containing 2 mM EDTA and washed twice with Eagle's/0.1%BSA before binding experiments were initiated. (A) *Ligand binding* was performed for 3 hours at 4°C in the presence of 2 ug/mL ^125^I-ASOR. After the 3 hours, cells were washed 4-5 times with Eagle's medium, and amounts of radioactive ligand bound were determined. Nonspecific binding (less than 10% of specific binding) was determined in the presence of 100-fold excess unlabeled ligand. (B) *Antibody binding* was also performed at 4°C, but in this case, the cells were incubated with primary antibody (1 : 100 final dilution) for 1 hour, washed, and then incubated with ^125^I-protein A for 1 hour [8]. Cells were then washed, and radioactivity associated with the cells was determined. Nonspecific binding was determined in the presence of nonimmune serum.

### 2.4. Internalization and Degradation of ASOR

 Internalization and degradation of ^125^I-ASOR was determined over a time course of 5 hours. ^125^I-ASOR was added to cell cultures at a final concentration of 2 ug/mL, and at the indicated times, the flasks were placed on ice and the cells removed from the plate by the addition of 2 mM EDTA to the wells. Degradation products in the media were determined by the presence of acid-soluble radioactivity, while internalized ligand was represented by radioactivity in the cell pellet.

### 2.5. General

Results are expressed as fmoles ASOR bound, internalized or degraded per million cells. A normalized value of 11 ug DNA per million cells was used for these calculations [9]. Statistical analysis was determined using the Student's *t* test. A probability of 0.05 or less was considered significant.

## 3. Results

**Figure 1:**
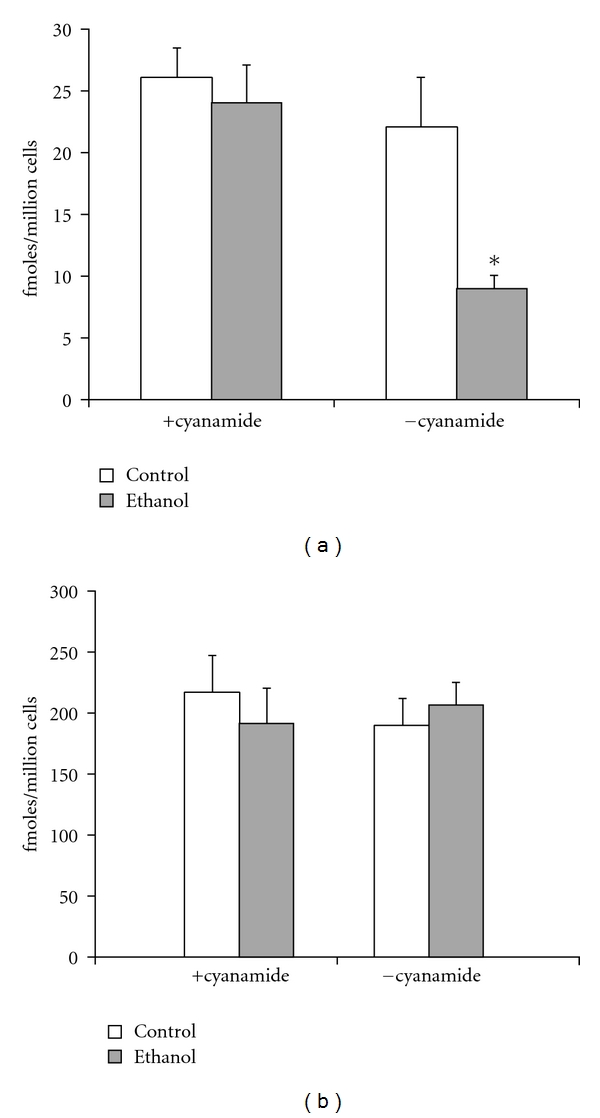
(a) Effects of ethanol (25 mM) and cyanamide (0.1 mM) on binding of ^125^I-ASOR to HAD cells treated for 7 days. HAD cells were grown to confluence and treated as described in Section 2. Ligand binding was performed at 4°C for 3 hours and non-specific binding was determined in the presence of 100-fold excess unlabeled ligand. Data are presented as means ± SEM for 6–10 sets of cells. Legends: control: HAD cells cultured in the absence of added ethanol; ethanol: HAD cells cultured in the presence of 25 mM ethanol. Values significantly different from controls are indicated, *P < 0.05. (b) Effects of ethanol administration (25 mM) and cyanamide (0.1 mM) on binding of anti-ASGP-R antibody to HAD cells. Cell cultures were obtained from the same conditions as in Figure 1(a). Binding of antibody was determined by the ability of cells to bind a polyclonal antihuman ASGP-receptor antibody, followed by radiolabeled detection of ^125^I-protein A. Data are presented as means ± SEM for 6–10 experiments. Legends: control: HAD cells cultured in the absence of added ethanol; ethanol: HAD cells cultured in the presence of 25 mM ethanol.

Initially, we examined the ability of the ligand, ASOR, to bind to the ASGP-R after culturing HAD cells in the presence of 25 mM ethanol for 7 days either in the presence or absence of the aldehyde dehydrogenase inhibitor, cyanamide. These data are shown in Figure 1. The addition of ethanol alone to the growth media did not result in changes in ASOR binding. In the presence of cyanamide, which increases acetaldehyde concentrations to uM levels in these cells [9], the binding was significantly impaired by 55% (Figure 1(a)). These data show that there was no measurable impairment in ligand binding in the presence of ethanol alone in this series of experiments, but that during ethanol metabolism and inhibition of aldehyde dehydrogenase activity ligand binding was significantly decreased. Monitoring acetaldehyde levels revealed that acetaldehyde levels in cells cultured in the presence of ethanol and cyanamide peaked at approximately 150 uM at 36 hours and then gradually declined.

We next examined the ability of a polyclonal antibody against human ASGP-R to bind to the cells under these experimental conditions. No differences in antibody binding were seen in the HAD cell populations, regardless of treatment conditions (Figure 1(b)). Thus, the levels of the receptor protein are unchanged in these cultures. The data presented here, along with that from Figure 1(a), indicate an inactivation of the receptor as a result of acetaldehyde-induced inhibition of ligand binding in the ethanol-treated cultures containing cyanamide (a powerful inhibitor of aldehyde dehydrogenase). This inactivation phenomenon is similar to that observed in rat hepatocytes after ethanol administration for 1-2 weeks [8].

**Figure 2:**
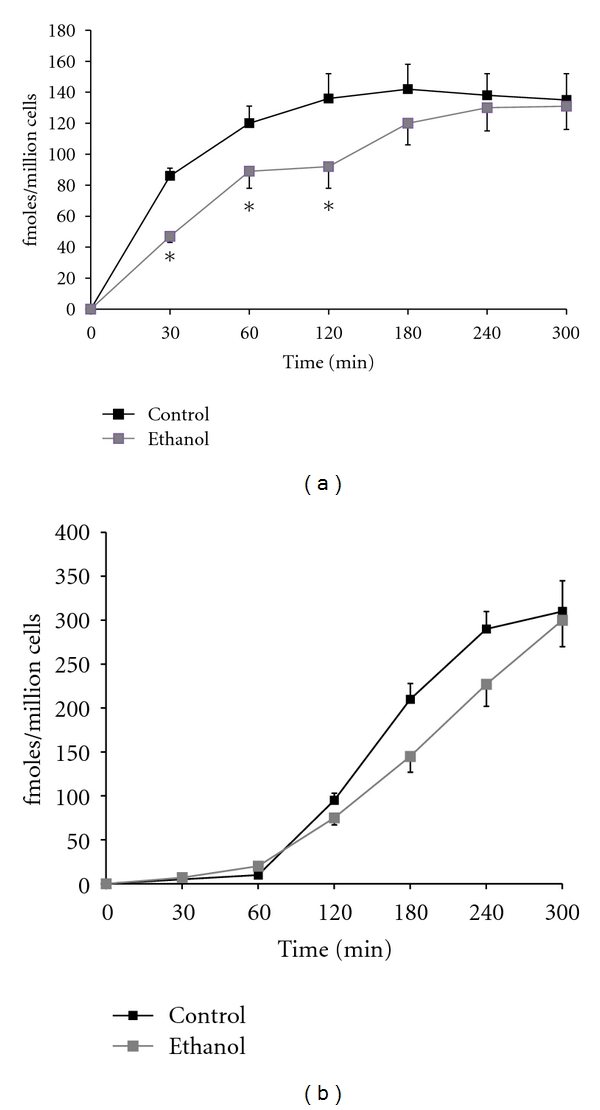
Kinetics of internalization (a) and degradation (b) of ^125^I-ASOR in HAD cells after 7 days of treatment with 25 mM ethanol. Cells were treated with (□) or without (■) ethanol for 7 days as previously described. At the end of the treatment period, cells were incubated with Eagles medium containing 1% BSA and 2 ug per mL iodinated ASOR. At the indicated times, duplicate plates were removed from the 37°C incubator, the cells were removed from the plates with 2 mM EDTA, and internalization (pellet associated, a) and degradation (acid-soluble media radioactivity, b) were determined. Data are presented as fmoles internalized or degraded per million cells and are means ± SEM for 6–10 experiments. Values significantly different from controls are indicated, *P < 0.05.

We also examined the effects of ethanol oxidation on the ability of the asialoglycoprotein receptor to internalize ligand. The results indicated that internalization of ASOR was significantly impaired early in the time course of internalization (the first 150 min, Figure 2(a)), and that the addition of cyanamide further potentiated this impairment (Figure 2(b)). Since cyanamide alone (in the absence of ethanol) did not alter internalization of ASOR, the results implicate a role for acetaldehyde in the impaired internalization in these cells.

**Figure 3:**
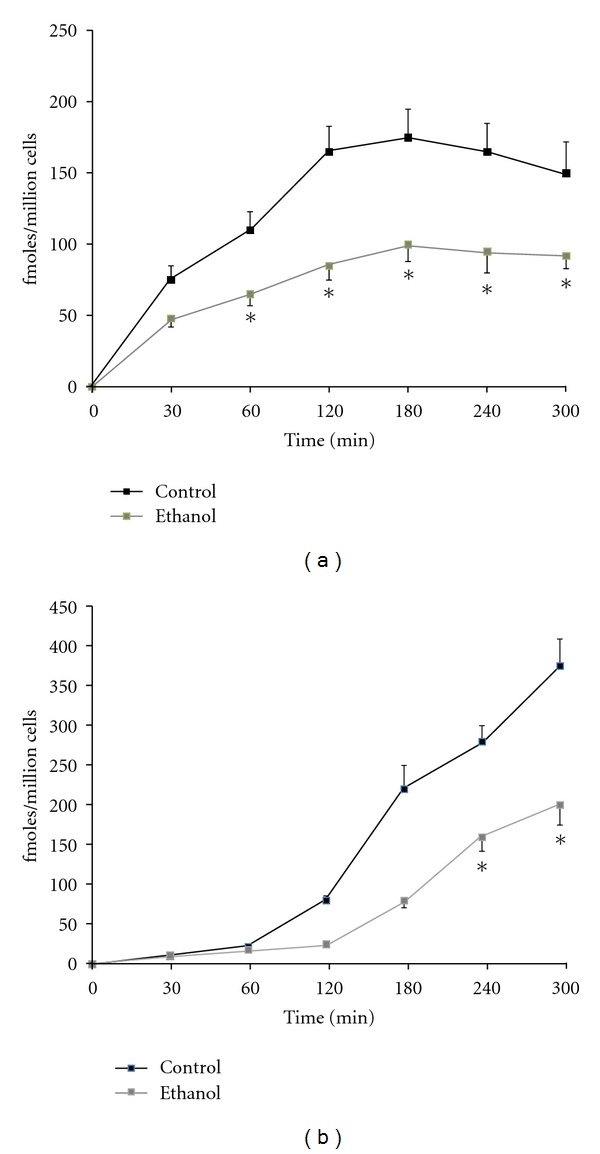
Effects of cyanamide on kinetics of internalization (a) and degradation (b) of ^125^I-ASOR in HAD cell cultures grown in the presence (□) or absence (■□) of 25 mM ethanol. Internalization (a) and degradation (b) of ASOR were determined as described in Figure 3. Data are presented as fmoles ASOR degraded or internalized per million cells and are means ± SEM for 4-5 separate experiments. Values significantly different from controls are indicated, *P < 0.05.

We also investigated the degradation of ASOR in these cell populations. In the presence of ethanol alone, degradation of ligand was unaltered over the 150 min time course of the experiment (Figure 3(a)). However, in the presence of cyanamide in the culture medium, the degradation was decreased over the 5-hour course (Figure 3(b)). Again, these data implicate the direct involvement of acetaldehyde in these dysfunctions and further support the suggestion that acetaldehyde mediates many of the impairments associated with ethanol-induced hepatic cytotoxicity.

## 4. Discussion

We have previously reported the establishment of a recombinant hepatic cell line (HAD) that stably expresses alcohol dehydrogenase and oxidizes ethanol to acetaldehyde. During ethanol metabolism, ligand binding to the ASGP-R was shown to be decreased, and the impairment was alleviated upon the addition of an alcohol dehydrogenase inhibitor, pyrazole. These results suggested that ethanol metabolism was necessary for the observed impairments in ligand binding. Data presented in this study demonstrate that after 7 days of ethanol exposure in HAD cells, ligand internalization is also impaired. These impairments were most apparent in the early times of internalization of the ligand. Upon addition of cyanamide, an aldehyde dehydrogenase inhibitor known to increase acetaldehyde levels in cells actively metabolizing ethanol, the impairments in internalization of ASOR were potentiated dramatically. In addition, degradation of ASOR and binding to its receptor were both significantly decreased when the cells were incubated with ethanol and cyanamide, but not with ethanol alone. The presence of cyanamide alone, in the absence of ethanol, did not alter any aspect of endocytosis tested. Additionally, we have demonstrated that higher levels of acetaldehyde increase the severity of other ethanol-metabolism-mediated impairments [13]. Taken together these data from the present study as well as previous data implicate an important role for acetaldehyde in the ethanol-induced impairments in endocytosis in HAD cells.

These findings are important and relevant to our understanding and examination of ethanol-induced alterations in protein trafficking in the liver. It has been suggested that the production of acetaldehyde may be responsible for some of the hepatic impairments attributed to the oxidation of ethanol [14, 15], and the formation of acetaldehyde-protein adducts could be the mechanism by which alcohol ultimately damages cells [16, 17]. Although acetaldehyde is strongly implicated as a mediator of the ethanol-induced dysfunction in hepatic protein secretion, the role of acetaldehyde in impaired RME and signal transduction has not been established. In addition, this has been difficult to examine mechanistically in a culture system, since the ability of hepatocytes to efficiently metabolize ethanol, as well as many other liver-specific functions, are rapidly lost in culture [18]. The development of a hepatic cell line which is capable of oxidizing ethanol, along with results presented in this study showing a direct involvement of acetaldehyde in ethanol-impaired RME in these cells, allows a differentiation between the hepatotoxic effects of ethanol itself and its oxidation products. This is the first report for a direct effect of acetaldehyde on a continuous endocytic process, such as internalization and degradation of ligands by RME. The results of these studies should aid in an examination of the hypothesis that the production of acetaldehyde and its presence in the cell could lead to the formation of acetaldehyde-protein adducts and through their accumulation eventually cause hepatic dysfunction.

In conclusion, these studies show a direct involvement for ethanol metabolism and subsequent acetaldehyde production during the impaired receptor-mediated endocytosis which occurs after ethanol administration. Use of the HAD cells allows us to have the ability to examine the mechanism(s) which are responsible for the ethanol-induced impairments in liver cell function, which we feel are related to the progression of alcoholic liver injury. Future work with the HAD cells will aid in the elucidation of ethanol's effects on altered endocytosis, signalling events, and protein trafficking events in the liver. In addition, we can now examine the role of acetaldehyde in the impairments.

## References

[1] Casey CA, Kragskow SL, Sorrell MF, Tuma DJ (1987). Chronic ethanol administration impairs the binding and endocytosis of asialo-orosomucoid in isolated hepatocytes. *Journal of Biological Chemistry*.

[2] Casey CA, Kragskow SL, Sorrell MF, Tuma DJ (1989). Ethanol-induced impairments in receptor-mediated endocytosis of asialoorosomucoid in isolated rat hepatocytes: time course of impairments and recovery after ethanol withdrawal. *Alcoholism*.

[3] Casey CA, Kragskow SL, Sorrell MF, Tuma DJ (1990). Effect of chronic ethanol administration on total asialoglycoprotein receptor content and intracellular processing of asialoorosomucoid in isolated rat hepatocytes. *Biochimica et Biophysica Acta*.

[4] Casey CA, Tuma DJ, LeBouton AV (1993). Receptors and endocytosis. *Molecular and Cell Biology of the Liver*.

[5] Casey CA, Kragskow SL, Sorrell MF, Tuma DJ (1991). Zonal differences in ethanol-induced impairments in receptor-mediated endocytosis of asialoglycoproteins in isolated rat hepatocytes. *Hepatology*.

[6] McCashland TM, Tuma DJ, Sorrell MF, Casey CA (1993). Zonal differences in ethanol-induced impairments in hepatic receptor binding. *Alcohol*.

[7] Dalke DD, Sorrell MF, Casey CA, Tuma DJ (1990). Chronic ethanol administration impairs receptor-mediated endocytosis of epidermal growth factor by rat hepatocytes. *Hepatology*.

[8] Tworek BL, Tuma DJ, Casey CA (1996). Decreased binding of asialoglycoproteins to hepatocytes from ethanol-fed rats: consequence of both impaired synthesis and inactivation of the asialoglycoprotein receptor. *Journal of Biological Chemistry*.

[9] Clemens DL, Halgard CM, Miles RR, Sorrell MF, Tuma DJ (1995). Establishment of a recombinant hepatic cell line stably expressing alcohol dehydrogenase. *Archives of Biochemistry and Biophysics*.

[10] Fallon RJ, Schwartz AL (1988). Asialoglycoprotein receptor phosphorylation and receptor-mediated endocytosis in hepatoma cells. Effect of phorbol esters. *Journal of Biological Chemistry*.

[11] Clemens DL, Halgard CM, Cole JR, Miles RM, Sorrell MF, Tuma DJ (1996). Impairment of the asialoglycoprotein receptor by ethanol oxidation. *Biochemical Pharmacology*.

[12] Knowles BB, Howe CC, Aden DP (1980). Human hepatocellular carcinoma cell lines secrete the major plasma proteins and hepatitis B surface antigen. *Science*.

[13] Clemens DL, Forman A, Jerrells TR, Sorrell MF, Tuma DJ (2002). Relationship between acetaldehyde levels and cell survival in ethanol-metabolizing hepatoma cells. *Hepatology*.

[14] Jennett RB, Tuma DJ, Sorrell MF, Popper H, Schaffner F (1990). Effects of acetaldehyde on hepatic proteins. *Progress in Liver Disease*.

[15] Lieber CS (1988). Metabolic effects of acetaldehyde. *Biochemical Society Transactions*.

[16] Sorrell MF, Tuma DJ (1985). Hypothesis: alcoholic liver injury and the covalent binding of acetaldehyde. *Alcoholism*.

[17] Tuma DJ, Hoffman T, Sorrell MF (1991). The chemistry of acetaldehyde-protein adducts. *Alcohol and Alcoholism*.

[18] Zakhari S (2006). Overview: how is alcohol metabolized by the body?. *Alcohol Research and Health*.

